# Novel Strategies to Improve the Cardioprotective Effects of Cardioplegia

**DOI:** 10.2174/011573403X263956231129064455

**Published:** 2024-01-24

**Authors:** Estefanie Osorio-Llanes, Jairo Castellar-López, Wendy Rosales, Yuliet Montoya, John Bustamante, Ricardo Zalaquett, Roberto Bravo-Sagua, Jaime A. Riquelme, Gina Sánchez, Mario Chiong, Sergio Lavandero, Evelyn Mendoza-Torres

**Affiliations:** 1 Faculty of Exact and Natural Sciences, Grupo de Investigación Avanzada en Biomedicina, Universidad Libre Barranquilla, Atlantico, Colombia;; 2 Grupo de Dinámica Cardiovascular (GDC), Escuela de Ciencias de la Salud, Universidad Pontificia Bolivariana, Medellin, Colombia;; 3 Department of Cardiovascular Diseases, Faculty of Medicine, Universidad Finis Terrae - Clínica Las Condes, Santiago, Chile;; 4 Advanced Center for Chronic Diseases (ACCDiS), Faculty of Chemical and Pharmaceutical Sciences & Faculty of Medicine, Universidad de Chile, Santiago, Chile;; 5 Laboratorio OMEGA, INTA, University of Chile, Santiago, Chile;; 6 Physiopathology Program, Institute of Biomedical Sciences (ICBM), Faculty of Medicine, University of Chile, Santiago, Chile;; 7 Department of Internal Medicine (Cardiology Division), University of Texas Southwestern Medical Center, Dallas, Texas, USA;; 8 Faculty of Health Sciences, Grupo de Investigación Avanzada en Biomedicina, Universidad Libre Seccional Barranquilla, Barranquilla, Colombia

**Keywords:** ischemia/reperfusion, cardiovascular disease, cardioplegia, cardioprotection, cardiac surgery, angiotensins

## Abstract

The use of cardioprotective strategies as adjuvants of cardioplegic solutions has become an ideal alternative for the improvement of post-surgery heart recovery. The choice of the optimal cardioplegia, as well as its distribution mechanism, remains controversial in the field of cardiovascular surgery. There is still a need to search for new and better cardioprotective methods during cardioplegic procedures. New techniques for the management of cardiovascular complications during cardioplegia have evolved with new alternatives and additives, and each new strategy provides a tool to neutralize the damage after ischemia/reperfusion events. Researchers and clinicians have committed themselves to studying the effect of new strategies and adjuvant components with the potential to improve the cardioprotective effect of cardioplegic solutions in preventing myocardial ischemia/reperfusion-induced injury during cardiac surgery. The aim of this review is to explore the different types of cardioplegia, their protection mechanisms, and which strategies have been proposed to enhance the function of these solutions in hearts exposed to cardiovascular pathologies that require surgical alternatives for their corrective progression.

## 1 INTRODUCTION

Cardiovascular diseases are the leading cause of morbidity and mortality worldwide [1]. Many cardiovascular pathologies require surgical interventions to control their progression [2]. However, the myocardial damage that occurs due to these interventions involves the need to put the heart in asystole, which is achieved with the use of cardioplegic solutions for rapid cardiac arrest [3-5]. According to Hausenloy *et al.*, patients undergoing coronary artery bypass grafting (CABG) are at higher risk of worse clinical outcomes due to their co-morbidities [6]. Heart coronary bypass surgery has become a good option; however, this presents some limitations, such as hemodynamic instability and incapacity to find a deeply embedded target vessel, making CABG a better alternative in these cases [7, 8].

Myocardial protection is essential in cardiac surgery and refers to several techniques used to preserve ventricular function by increasing the heart's ability to withstand ischemic periods while providing a bloodless and stationary operating field [8, 9]. In addition to timely restoration of blood flow, cardioprotection is important to attenuate the myocardial damage caused by ischemia, decrease the infarct size and improve the patient prognosis [10].

Ischemia/reperfusion (I/R) episodes may damage the heart undergoing surgery. After an ischemic episode, cardiac cells recover their function with the blood supply and oxygen levels of the heart under normal conditions. Still, when reperfusion is induced paradoxically, many cardiomyocytes die, leading to extensive tissue damage [11]. Several studies have been dedicated to looking for mechanisms that protect the myocardium against I/R damage [12, 13]. The purpose of cardioplegic solutions is to stop very quickly the mechanical and electrical activity of the heart, reduce the metabolic activity of the myocardium during periods of ischemia, slow down intracellular acidosis and reduce the excess Ca^2+^ in the myocyte cytosol [14-16]. Cardiac arrest was first applied in 1955, and tactics for cardioplegia management have evolved to combat clinical problems since then. Each of these strategies provides a tool to counter myocardial damage [17-19].

As the complexity of cardiac surgeries and the need for myocardial protection increase, the need for the development of new cardioplegic solutions also increases. In recent years, this need has benefited the production of novel solutions, such as crystalloid or blood cardioplegic solutions, with different administration directions, such as antegrade or retrograde cardioplegia, and cardioplegia that depends on the temperature of administration and single or multidose infusion [8].

The cardioplegic solutions of St. Thomas, Burckberg, del Nido, and Custodiol are the most used and popular in cardiovascular surgeries, and del Nido is one of the most efficient [20, 21]. Dr. del Nido and colleagues created a single-dose cardioplegic solution, a combination of long-acting blood and crystalloids elements, especially for pediatric patients [22]. The addition of polarizing agents such as lidocaine is thought to decrease energy consumption, and the presence of Ca^2+^-competing ions such as Mg^2+^ at optimum concentration reduces intracellular Ca^2+^ concentration, thus preventing cell damage [23].

Cardioplegia is used, almost universally, to provide a clean and bloodless space that is critical for surgical precision and myocardial protection during the ischemic period. However, the tactics for the management of cardioplegia must evolve to prevent clinical problems, and each strategy must provide a tool to counteract the myocardial damage [24]. This review aims to present the different types of cardioplegia available and the strategies that have been proposed to protect the heart during and after cardioplegia.

## 2 GENERAL ASPECTS OF CARDIOPLEGIA

**Fig. (1):**
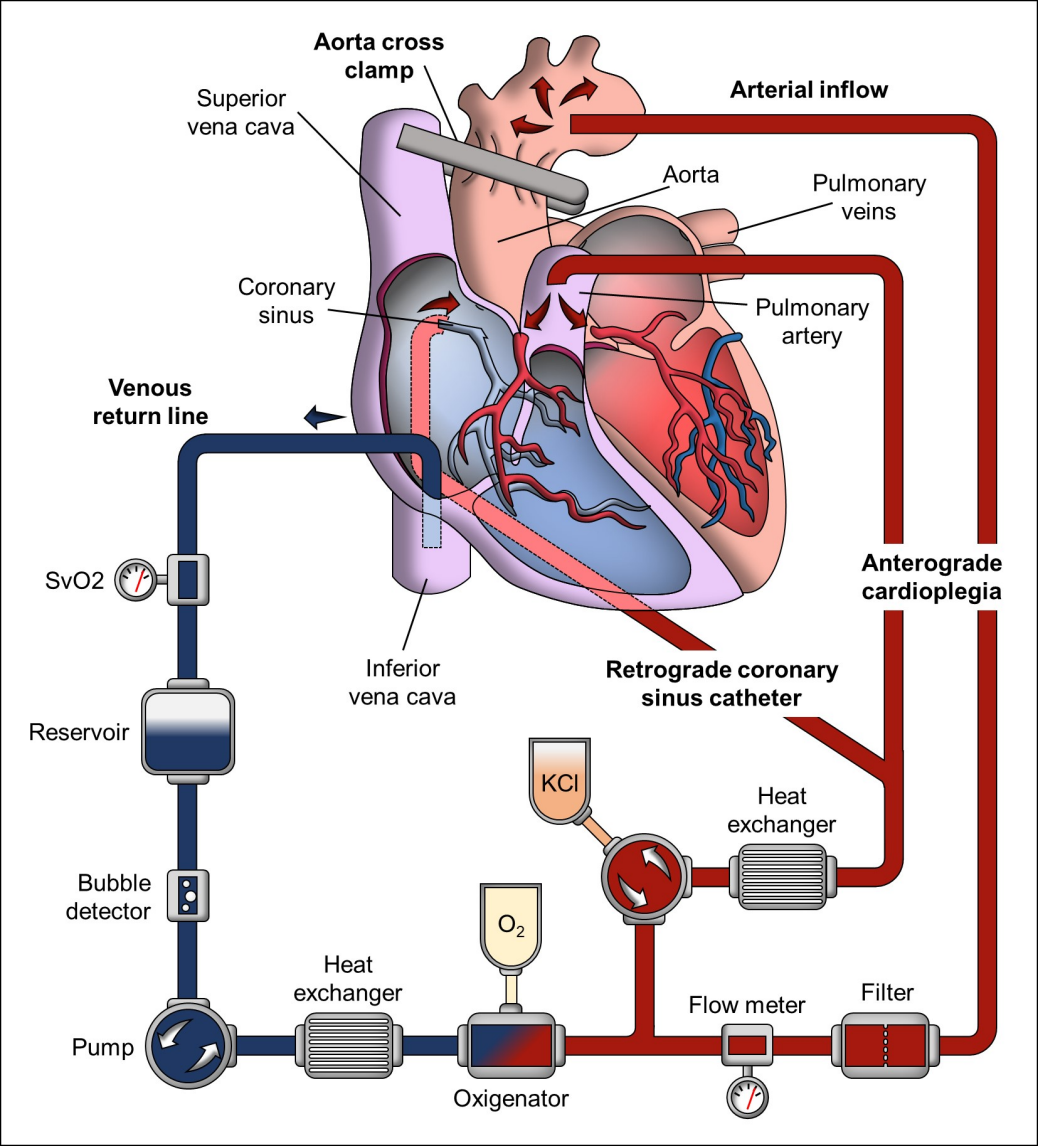
Extracorporeal circuit diagram.

Cardioplegia is used in open heart surgery, which requires extracorporeal circulation, or cardiopulmonary bypass (CPB), for which a heart-lung machine, called a “pump,” is used (Fig. **1**) [25]. The blood may be drained by either cannulation of the right appendage (*i.e.,* coronary or aortic surgery) or venae cavae (*i.e.*, mitral surgery, congenital surgery, or tricuspid replacement). In the latter case, tourniquets are placed around the cannulas inserted into the venae cavae; otherwise, the operative field is flooded with blood, and CPB is stopped by the entry of air that prevents venous drainage. The oxygenated blood must return from the heart-lung machine to the body, which is also known as “perfusion.” Thus, the most used site for cannulation is the distal ascending aorta. In case this is not possible, the femoral artery or axillary artery can be cannulated [25, 26].

In CPB, the pump takes over the function of the heart and lungs. The heart maintains electro-mechanical activity but does not eject blood. In these conditions, with the heart beating, some procedures can be done in the right heart or in the epicardial coronary arteries. In cases where it is necessary to stop the heart (*i.e.,* aortic valve surgery, ascending aorta, or coronary bypass), the aorta is cross-clamped immediately proximal to the aortic cannula. The heart will stop after a few minutes due to ischemia, starting the “ischemic period”. This is how open-heart surgery was performed for a long time until “cardioplegia” was introduced in the 1970s [27].

Cardioplegia aims to quickly stop the heart to decrease cardiac work and, consequently, oxygen consumption. Most cardioplegic solutions are infused at 4°C, to decrease the heart's metabolism. Antegrade cardioplegia is infused into the aortic root, placing a fine catheter into the ascending aorta. As the distal ascending aorta is cross-clamped and the aortic valve is competent, cardioplegia has no other path than the coronary arteries. If the aortic valve is insufficient or the ascending aorta is opened, cardioplegia may be infused into the coronary ostia by directly cannulating them. Retrograde cardioplegia is infused into the coronary sinus. To achieve this, the coronary sinus is cannulated with a specially designed cannula through the right atrium, fixing it with a purse string. If the right atrium has been opened, the coronary sinus is cannulated directly, fixing the cannula with a purse string in the ostium of the coronary sinus [28, 29].

Despite the aorta being occluded and the venous blood drained by the pump, blood reaches the left heart through the bronchial circulation, which passes through the pulmonary veins to the atrium and left ventricle, aorta, and coronary arteries. In aortic surgery, a left atrioventricular catheter is placed through the right superior pulmonary vein. The heart can beat again as the vent is not completely effective and because there is non-coronary circulation that perfuses the heart. For this reason, most cardioplegia protocols are multidose, repeating cardioplegia every 15 to 30 minutes. Avoiding this repetition is possible with Custodiol or del Nido cardioplegia [30, 31].

Cardioplegia is the cornerstone of cardioprotection during cardiac surgery. However, despite 60 years of experience in this method, it has undergone few innovations, except for blood cardioplegia [13]. Cardioplegic solutions are accompanied by stabilizing substances that help improve cardiac function. Most of the components are electrolytes at different concentrations, active ingredients that induce pharmacological activity and may present cardioprotective effects. Studies have shown that the application of a good cardioplegic solution would considerably improve the long-term prognosis for patients undergoing cardiovascular surgery [32]. Finding the correct cardioplegia depends also on the type of the patient and its clinical records. Indeed, the risk of death after cardiovascular surgery is elevated in children and adult patients with several comorbidities since their hearts are susceptible to increased damage after an I/R injury episode [33].

The basic principles of any cardioplegia solution are (1) a rapid and effective stop in diastole to keep the myocardium relaxed and minimize adenosine triphosphate (ATP) usage; (2) a protection phase that can delay the time in which irreversible myocardial damage due to ischemia occurs and limit the damage due to reperfusion, and (3) a reversibility phase, in which the effects of cardioplegia must be quickly reversible to ensure a proper recovery of cardiac function. These substances are expected to have low toxicity, with a short half-life and without harmful effects on other organs [34].

## 3 TYPES OF CARDIOPLEGIA

### 3.1 Crystalloid Cardioplegia

Crystalloid cardioplegia solutions were introduced clinically in the mid-1960s [35]. Typically, these are delivered at low temperatures (‘cold’ crystalloid cardioplegia), which provides myocardial protection mainly by hypothermia and electromechanical arrest, prolonging the myocardium’s tolerance to ischemia. Crystalloid cardioplegia can contain low or no Na^+^ or Ca^2+^ (HTK solution). On the contrary, these solutions can contain high concentrations of Na^+^, Ca^2+^, and Mg^2+^ (STH solution) [16]. Potential disadvantages of crystalloid cardioplegia are dilutional hyponatremia and reduced intraoperative hematocrit, which may result in the need for postoperative blood transfusions and longer hospital admissions [36].

Cold crystalloid cardioplegia associated with moderate hypothermia offers cardioprotection during low-flow periods, and it is easy to use [24, 37]. However, the reduction of mitochondrial respiration caused by hypothermia has an adverse outcome on the functional and metabolic recovery of the heart and leads to delayed recovery. Nardi *et al*. observed better myocardial protection with the use of cold rather than warm cardioplegia in patients with comorbidities such as left ventricular hypertrophy and aortic valve disease [37].

### 3.2 Brettschneider’s Histidine-Tryptophan-Ketogluta-rate (HTK) Cardioplegia Solution

HTK solution is commercially available as Custodiol and is widely used for organ preservation in transplant surgery [38, 39]. Custodiol is an intracellular crystalloid cardioplegic solution (Table **1**). According to Edelman *et al*., Custodiol is an attractive solution for surgeons because it is administered as a single dose and allows protection for a period of up to three hours [39]. Histidine acts as a buffer, counteracting the effects of acidosis secondary to the accumulation of anaerobic metabolism products during the period of ischemia; ketoglutarate improves ATP synthesis during reperfusion; tryptophan that stabilizes the cell membrane, and finally, mannitol reduces cell edema and neutralizes free radicals [40]. Bretschneider’s HTK solution caused less severe endothelial injury than cold blood cardioplegia [41].

### 3.3 St Thomas' Hospital (STH) Cardioplegic Solution

STH solution 1 (STH-1) was developed in the early 1970s by Hearse *et al*. [42]. This solution extended considerably the tolerance during ischemic arrest under both normothermic and hypothermic conditions. Years later, a novel formulation was developed, which became available with the name of STH solution 2 (STH-2 or Plegisol). The main characteristics of STH-2, compared with STH-1, are a small reduction in the Na^+^ and K^+^ concentration, a reduction in Ca^2+^ content by 50%, the absence of procaine and the inclusion of bicarbonate [42]. STH-2 continues to be appealing for minimally invasive cardiac surgery, having cardioprotective applications for up to two hours [43].

### 3.4 Blood Cardioplegia

Blood cardioplegia facilitates rapid cardiac arrest in an oxygenated environment. The intermittent reoxygenation of the myocardium negates the need for additive anaerobic substrates such as glucose [44]. Barner mentions how warm blood cardioplegia reduces the risk of hemodilution as it approximates normal physiology [44]. The reperfusion of ischemic myocardial tissue results in the production of reactive oxygen species (ROS), which may worsen myocardial damage. Blood contains endogenous oxygen radical scavengers that protect against oxidative stress [45]. Rinne *et al*. demonstrated that spontaneous resumption of sinus rhythm occurred more frequently with blood cardioplegia when compared to crystalloid [46].

### 3.5 Warm Cardioplegia

Warm cardioplegia theoretically avoids the myocellular injury inflicted by hypothermia. Lichtenstein *et al*. studied the outcomes of high-risk patients who underwent CABG with cold and warm cardioplegia. There was a significantly lower incidence of perioperative myocardial infarction and prevalence of low output syndrome with warm cardioplegia. Furthermore, 99.2% of patients with warm cardioplegia resumed spontaneous sinus rhythm following aortic cross-clamp removal as compared to only 10.5% of patients with cold cardioplegia [24, 47]. Fan *et al*. conducted a meta-analysis of 41 randomized controlled trials comparing the outcomes of 5,879 patients. The incidence of myocardial infarction, low output syndrome, atrial fibrillation, stroke, and intra-aortic balloon pump support were similar to cold and warm cardioplegia. However, a decrease in postoperative enzyme release and improvement in postoperative cardiac index with warm cardioplegia was observed [48]. Warm blood cardioplegia provides up to 2 to 3 hours of myocardial preservation [49].

### 3.6 Del Nido Cardioplegia

Del Nido solution was developed in 1995 as a single-dose cardioplegic solution for use in congenital heart defect corrective surgery. Del Nido is an extracellular solution with a 1:4 blood crystalloid ratio. It contains lidocaine (140 mg/L), mannitol, MgSO_4_ and a lower concentration of Ca^2+^ compared to other cardioplegic solutions [50]. This solution benefits a safe ischemic process time through a reduction in Ca^2+^ influx and preservation of intracellular phosphate and pH. During cardiac arrest, a small number of Na^+^ and Ca^2+^ channels remain active with membrane potential depolarization. The lidocaine blocks Na^+^ channels and the Mg^2+^ acts as a Ca^2+^ antagonist, reducing diastolic intracellular Ca^2+^ concentration [51]. Importantly, CPB and cross-clamp period were lower with del Nido solution. This can be attributed to the reduced time for cardioplegia administration associated with Del Nido, which is commonly used in pediatric cardiac surgery [52]. Del Nido cardioplegia provides myocardial protection of 3 hours in cardiac arrest during aortic clamping [53]. However, there is a need for further prospective randomized trials to address its safety in adults thoroughly.

### 3.7 Cardi-Braun's Cardioplegia

This cardioplegia is commercially distributed as a “Cardi-Braun Maintenance solution for infusion”. Cardi-Braun's cardioplegia is induced through hot and cold inductions, followed by maintenance and, finally reperfusion. However, it is not the only one available on the market [54, 55]. Table **1** shows the composition of the cardioplegic solutions.

## 4 CARDIOPROTECTION MECHANISMS OF CARDIOPLEGIC SOLUTIONS

Myocardial damage is one of the significant causes of mortality in cardiovascular surgery [55]. Therefore, myocardial protection is a critical approach to prevent or reduce myocardial complications that occur during and after cardiac surgery [56].

Cardioplegic solutions protect the myocardium against I/R injury by abolishing the generation and propagation of action potentials and the resulting contractions that are responsible for most of the metabolic activity of the heart, thus mitigating myocardial injury [56]. Elderly patients undergoing cardiac surgery have a significantly higher risk of cardiac dysfunction because older hearts are more susceptible to injury after periods of ischemia that occur during cardiovascular surgery. The mechanism for this intolerance to ischemia in the senescent myocardium is related to changes in Ca^2+^ homeostasis that result in higher intracellular Ca^2+^ levels during ischemia [56]. On the other hand, an immature myocardium has unique structural and functional characteristics compared to a mature myocardium. These characteristics influence the protective responses of the pediatric myocardium to ischemia during cardiac surgical procedures. However, even if the pediatric myocardium is resistant to ischemia, cardiac surgery in these patients almost always occurs under stressors that increase intracellular Ca^2+^ levels, generating significant cardiac damage [57]. Cardioplegia is crucial for its cardioprotective use in the hearts of elderly and pediatric patients. However, new advances have been directed to these protection solutions to improve the effects of the cardioplegic solution and guarantee cardiac preservation [58].

### 4.1 Proposed Mechanisms for the Improvement of Cardioplegia

The ideal method of myocardial protection during open-heart surgery continues to be among the most debated areas in cardiac surgery. The two goals of the cardiac surgical practice are technical excellence and the safety of cardiac function. However, the antegrade mode during which the cardioplegia solution is infused through the aortic root remains the preferred method in many centers due to its safety and simplicity [59].

Ischemic preconditioning has been used in cardiac surgery as a cardioprotective strategy. This technique has generated a decrease in ventricular arrhythmias in patients in intensive care [60]. However, more studies are required to extend its application. Remote ischemic preconditioning preserved mitochondrial respiration and prevented upregulation of miR-1, reduced atrial fibrillation, and protection from I/R in the right atrium in patients undergoing CABG [61].

Veres *et al*., prepared a preclinical and experimental study to examine the efficacy of a new Custodiol cardioplegia. This solution differs from the one reviewed by Edelman *et al*. [41] in several compounds such as aspartate, arginine, alanine, glycine, sucrose, deferoxamine and the iron chelator LK- 614. As a result, *in vivo* endothelial function evaluated in dog’s hearts was improved with the new Custodiol Solution (Custodiol-N). Veres *et al*. concluded that Custodiol-N improved myocardial and endothelial function after CPB with hypothermic cardiac arrest [62].

Using only one type of cardioplegia in a unique or typical way is perhaps a practice of the past. Just as cardiac surgery has evolved, patients have evolved, and now patients who are present in the operating room have complex cardiac diseases. Optimal myocardial protection is an important aspect of cardiac surgery, and it may be necessary to integrate several methods to obtain the best results [24].

### 4.2 Molecules with Potential to Improve Cardioplegia

**Fig. (2):**
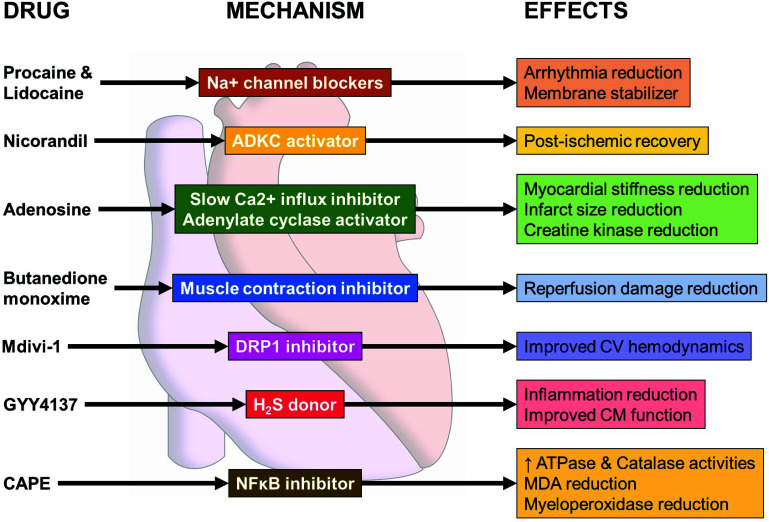
Proposed candidate molecules to improve cardioplegic solutions. Cardioplegia additives such as peptides, ADKC activators, and anesthetics decrease demands of the myocardium during I/R injury, reducing reperfusion damage, arrhythmias, and infarct size. A few of these molecules have the potential to stabilize cell membranes and protect cardiomyocytes, thereby enhancing myocardial protection.

Generally, cardioprotective strategies used to attenuate I/R injury following cardiac surgery have concentrated on the reduction in ischemic injury during the cardioplegic arrested period. These methodologies have focused attention on the alteration of cardioplegia constituents, the temperature of cardioplegia, and mostly on the method of how cardioplegia is delivered [63]. Several studies have been conducted to improve the effects of the cardioplegic solutions and to guarantee the preservation of cardiomyocytes both in ischemia and in reperfusion [64-67]. Current strategies in myocardial protection have advocated the use of cardioplegic solutions as a vector by which to introduce cardioprotective agents targeting specific mechanisms of ischemic reperfusion injury (Fig. **2**). However, the effectiveness of this strategy is predicated on the ability of the agent of interest to exert its therapeutic effects during the cardioplegic period or after that [68].

#### 4.2.1 Local Anesthetics

Among the components that have been evaluated to add to the cardioplegic solution are Na^+^ channel blockers that prevent the rapid depolarization of the cardiac cycle [35]. Local anesthetics such as lidocaine and procaine have been used to stabilize the membrane and have shown a decrease in arrhythmias in the postoperative period [69]. Lidocaine is a widely used local anesthetic and antiarrhythmic at lower concentrations [70]. This anesthetic has also shown neuroprotective [71], anti-inflammatory [72], and antioxidant effects [73]. The vasodilator effect of lidocaine may implicate nitric oxide (NO) [74] redox regulation [73] cAMP and cGMP signaling cascades that generate changes in cytosolic Ca^2+^ levels [75, 76]. As with others local anesthetics, procaine exerts effects at low concentrations on Na^+^ channels [77], which, in addition to its inhibitory effect on sarcoplasmic reticulum Ca^2+^ release channels, supports its antiarrhythmic action on the heart [78, 79].

#### 4.2.2 Activators of ATP-dependent K^+^ Channels (ADKC): Nicorandil

Nicorandil, an ADKC opener, and NO donor are currently used as a standard drug for the treatment of coronary artery disease in the clinical setting. ADKC openers have shown incidence of a cardioprotective effect against I/R injury in animal models [80-83] through mechanisms such as inhibiting apoptosis [82], reducing oxidative stress [83], reducing Ca^2+^ overload [84], and regulating mitochondrial membrane potential [85]. NO also plays a critical role in protection against I/R injury [86-88]. Takarabe *et al*. determined the cardioprotective efficacy of nicorandil in cardiac surgery using a surgically relevant 4-hour cardioplegic arrest model in isolated rabbit hearts. Pre-ischemia administration of nicorandil did not affect the recovery of developed pressure, however, administration of nicorandil after ischemia or before and after ischemia enhanced the recovery of developed pressure. The treatment with nicorandil attenuated I/R injury of the myocardium and coronary endothelium. These results indicate the potential of the use of nicorandil in the event of unexpected prolonged cardioplegic arrest [88].

Nicorandil improved the recovery of the post-ischemic contractile dysfunction of the heart and reduced the infarct size in perfused rat hearts subjected to global no-flow I/R [87]. As a single cardioplegic drug administered intermittently in cold blood, nicorandil preserved left ventricular contractility and myocardial energetics in pigs subjected to 1 hour of cold global ischemia [89]. Moreover, the CHANGE trial showed that nicorandil reduced the infarct size and improved left ventricular function in patients with ST-segment elevation myocardial infarction when this drug is administered prior to primary percutaneous coronary intervention [90].

Recently, a preclinical study determined that rats subjected to abdominal aortic constriction developed left ventricular hypertrophy. These rats showed higher frequency of ischemia-induced ventricular arrhythmias and increased cardiac interstitial concentrations of norepinephrine. Interestingly, treatment with nicorandil reduced the release of both norepinephrine and the incidence of ischemia-induced ventricular arrhythmias in hypertrophic hearts, likely by a mechanism that involves the opening of neuronal ADKCs [91]. Considering that nicorandil can increase NO as well as open K^+^-dependent ATP channels, it has been suggested that this drug can act as a multi-target therapeutic agent, thereby conferring potent cardioprotection [92, 93]. Accordingly, Liang *et al*., suggest that nicorandil, in combination with cardioplegic solutions, can decrease the severity of myocardial injury caused by I/R following cardiac arrest. Nicorandil treatment improves heart function, reduces myocardial necrosis, and inhibits myocardial apoptosis, all of which are important cardioprotective benefits [94]. Thus, considering the preclinical and clinical evidence, it stands to reason to speculate that nicorandil may be a powerful adjuvant to cardioplegia, but further research is needed to determine the optimal dosage and time exposure for nicorandil treatment effectiveness.

#### 4.2.3 Adenosine

Adenosine has a broad spectrum of cardioprotective properties when delivered as intravenous pretreatment. Adenosine as a pretreatment protects the heart *via* A1 and A2 receptors, producing smooth muscle relaxation by inhibition of slow Ca^2+^ influx and activation of adenyl cyclase mediated by A2 receptors in smooth muscle cells [95].

Jakobsen *et al*. showed that 1.2 mM adenosine instead of supranormal K^+^ in the cold (6°C) crystalloid cardioplegia improves post-cardioplegic left ventricular function and diminished myocardial injury in pigs subjected to aortic cross-clamp for 1 hour. The cold cardioplegia was given intermittently during aortic cross-clamp [96]. Ye & Chen determined the role of endogenous adenosine and endogenous adenosine receptors on ischemic preconditioning in aged rabbit hearts subjected to the Langendorff method. The experimental approaches they tested consisted of the administration of cardioplegic STH -II solution alone or accompanied by adenosine or adenosine receptor agonist (2-chloro-N(6)-cyclopentyladenosine). The combination of 2-chloro-N (6)-cyclopentadienone preconditioning and cold crystalloid cardioplegia had a considerable cardioprotective effect in aged rabbit hearts [97]. Cardiac function was preserved using the combination of 2-chloro-N(6)-cyclopentyladenosine, adenosine-enhanced ischemic preconditioning and cardioplegic STH-2 solution. This combination also prevented myocardial apoptosis, improved endothelial function, and protected the mitochondrial function of myocardial cells [97]. Furthermore, Law *et al*. showed that adenosine supplementation in cardioplegia attenuated C-terminal proteolysis of Troponin I and prevented its dissociation from myofilaments, indicating that the actions mediated by the adenosine receptor improve the functional recovery from myocardial arrest [98].

Jakobsen *et al.* have previously shown in experimental studies that substitution of potassium with adenosine (1.2 mmol/L) in cold crystalloid cardioplegia resulted in improved post-cardioplegic left ventricular systolic function and energetics, attenuated myocardial cell harm, and strides preservation of endothelial cells [96]. Furthermore, in a randomized clinical trial, sixty patients scheduled for elective CABG at the University Hospital of North Norway were randomized to receive either an STH solution (hyperkalemic group) or a solution in which hyperkalemia was replaced with 1.2 mmol/L adenosine. Adenosine in cold crystalloid cardioplegia gave more rapid cardiac arrest and showed cardioprotection and maintenance of hemodynamic parameters, together with an important reduction in the incidence of postoperative atrial fibrillation [99].

#### 4.2.4 Butanedione Monoxime

A reversible inhibitor of muscle contraction, butanedione monoxime, has been associated with a decrease in reperfusion damage when added to cardioplegic solutions in several experimental models of isolated rat hearts in the Langendorff system [100]. However, the systemic effects caused by this drug remain unsolved. Studies have used pyruvate, as well as a mixture of glutamate and aspartate, in the cardioplegic solution [101, 102]. However, the results obtained with these formulations have not generated a sufficient degree of confidence to extend their use in the operating room. Moreover, butanedione monoxime has been found to induce inhibition of mitochondrial respiration in adult mice cardiomyocytes by a mechanism that involves direct action on the electron transport chain, thus reducing cell viability [103]. Therefore, its translational value appears to be low, and caution should be taken before considering the use of butanedione monoxime in cardioplegia.

#### 4.2.5 Drp1 Inhibitors

Dynamin-related protein 1 (Drp1), a regulator of mitochondrial fission, plays a pivotal role in ROS generation, myocardial necrosis, and left ventricular dysfunction during I/R injury. Drp1 inhibition by Mdivi-1 administration during cardiopulmonary resuscitation following cardiac arrest preserved mitochondrial morphology and decreased oxidative injury in adult female C57BL/6 wild-type mice. Drp1 inhibition also improved cardiovascular hemodynamics following cardiac arrest [104]. Importantly, a study using adult pigs -an experimental model of high translational value- showed that Mdivi-1 did not reduce the infarct size or improve left ventricular function when administered at the beginning of reperfusion [105]. However, another study showed that hydralazine a drug used to reduce high blood pressure- can confer *in vitro*, *ex vivo* and *in vivo* cardioprotection [106]. Interestingly, molecular docking and surface plasmon resonance experiments suggest that hydralazine can bind to the GTPase domain of Drp1 and can dose-dependently inhibit its activity [106]. These findings suggest that hydralazine -an FDA-approved drug- is a therapeutic agent with high clinical value and may be repurposed to improve the protective effects of cardioplegia.

#### 4.2.6 Hydrogen Sulfide

Garcia *et al*. described how the hydrogen sulfide donor GYY4137 hydrogen sulfide (H_2_S) improved cardiomyocyte function in *ex vivo* Langendorff-perfused rat hearts subjected to cardiac arrest. Cardi-Braun^®^ and del Nido cardioplegia solutions supplemented with GYY4137 prevented apoptosis, ATP consumption, and oxidative stress through decrease of the S-(5-adenosyl)-L-methionine/S-(adenosyl)-L-homocy-steine ratio in perfused rat hearts, promoting the recovery of the electrophysiological status after cardiac arrest [107]. In Yorkshire, pigs underwent 1 h of crystalloid cardioplegia and CPB followed by 2 h of reperfusion. H_2_S improved the endothelium-dependent microvascular relaxation and attenuated caspase-independent apoptosis and autophagy [108].

#### 4.2.7 Caffeic Acid Phenethyl Ester (CAPE)

The administration of CAPE in cardioplegic STH cardioplegic solution N°2 improved the antioxidant defense system in isolated rat hearts mounted on a nonrecirculating type of Langendorff apparatus and subjected to cardiac arrest for 60 min with cardioplegic solution and then reperfused for 15 min. CAPE increased ATPase and catalase activities and reduced malonydealdehyde and myeloperoxidase activity, thus demonstrating that the administration of CAPE into cardioplegic solutions improves the antioxidant response of the rat heart during the I/R injury [109].

#### 4.2.8 Phosphodiesterase-5 Inhibitors: Vardenafil

Phosphodiesterase-5 (PDE5) inhibitors drugs, such as sildenafil, tadalafil, and vardenafil, are commonly used to improve erectile function, and the use of sildenafil has been approved for the treatment of pulmonary arterial hypertension. This molecule is also expressed in skeletal muscle, platelets, and myocardium [110]. Therefore, PDE5 inhibitors have a wide range of complex effects. Treatment with vardenafil improved myocardial and endothelial functions in a canine model after CB with cardioplegic arrest and extracorporeal circulation. Endothelium-dependent vasodilatory responses to acetylcholine were improved after the vardenafil administration [111]. According to research by Sahara *et al*., the PDE5 inhibitor vardenafil improved blood flow recovery, increased capillary collateral formation, and upregulated Vascular Endothelial Growth Factor (VEGF) and Hypoxia-Inducible Factor (HIF)-1 protein levels in wild-type C3H/He mice subjected to unilateral hindlimb ischemia. Furthermore, under hypoxia, vardenafil and cyclic guanosine monophosphate (cGMP) stimulated HIF-1 transactivation activity, indicating that HIF-1 is a target of vardenafil and cGMP in the context of ischemia-induced angiogenesis [112]. The phosphodiester bond of the cyclic nucleotides cyclic adenosine monophosphate (cAMP) and cGMP, which act as second messengers in numerous cellular functions, is hydrolyzed by PDE enzymes. As a result, depending on their substrate specificity, PDE inhibition can raise intracellular cAMP or cGMP levels, PDE-5 inhibitors might be ideal candidates for cardioprotection administered just before reperfusion [112].

#### 4.2.9 Molecules of the Counter-regulatory Renin-Angiotensin System (RAS) with Potential to Improve Cardioplegia

Recent studies have shown the positive effects of peptides from the counter-regulatory renin-angiotensin system in experimental models [113] and clinical trials [114]. Angiotensin-(1-9) (Ang-[1-9]) and angiotensin-(1-7) (Ang-[1-7]), produced by angiotensin converting enzyme 2 (ACE2) from Ang I and Ang II, respectively, have shown cardioprotective effects [115-117]. These peptides of endogenous origin prevented the development of cardiac hypertrophy in animal models [118-121] and decreased cardiac damage during I/R [117, 122]. Ang-(1-9) and Ang-(1-7) could be potential co-adjuvants for cardioplegic solutions and improve the postoperative result due to their cardioprotective properties.

##### 4.2.9.1 Angiotensin-(1-7)

Ang-(1-7) has shown antifibrotic, antihypertrophic, and anti-arrhythmogenic effects, as well as encouraging the production of NO [122, 123]. After the identification of the Mas receptor (MasR) as the main mediator of the effects of Ang-(1-7), the protective role of the Ang-(1-7) axis/MasR has been demonstrated against endothelial dysfunction, myocardial ischemia, I/R, ventricular remodeling, and heart failure [124, 125]. Miller *et al*. evaluated the effects of Ang-(1-7) on the cardiovascular system in aged animals and showed that Ang-(1-7) infusions improved the cardiometabolic function, systolic blood pressure, and insulin sensitivity [126].

Ang-(1-7) appears as an alternative to mitigate the cardiac damage induced by the dysregulation of intracellular Ca^2+^ that causes reperfusion arrhythmias because the effects of this peptide on the homeostasis of Ca^2+^ in cardiomyocytes subjected to simulated I/R [127]. Furthermore, Ferreira *et al*. showed that Ang-(1-7) reduced the incidence and duration of reperfusion arrhythmias in isolated rat hearts subjected to I/R [122]. Ang-(1-7) is a potential candidate to improve cardioplegic solutions thanks to its potential in the preservation of cardiac function and recovery after cardioplegic arrest.

##### 4.2.9.2 Angiotensin-(1-9)

Recent data have shown that Ang-(1-9) exerts protective effects on heart and blood vessels *via* Angiotensin II type 2 receptor (AT2R) [118, 119, 128-130]. In fact, Ang-(1-9) increased the secretion of atrial natriuretic peptide, a potent cardioprotective agent, in isolated perfused atria *via* AT2R-PI3K-Akt-NO [129]. In a study conducted by our research group, Ang-(1-9) decreased cell death by apoptosis and necrosis in cardiomyocytes subjected to I/R and improved ventricular function during reperfusion *via* AT2R/Akt in isolated rat hearts subjected to global ischemia for 30 minutes and reperfusion for 1 hour [130]. The propagation of injury during I/R events can be minimized by using cardioplegic solutions enriched with Ang (1-9) [131]. Fig. (**3**) shows the cardioprotective effects of Ang-(1-9) and Ang-(1-7) in preclinical models and that support their great potential to improve the cardioprotective effect of cardioplegic solutions.

Taken together, this evidence suggests that co-administration of these peptides with cardioplegic solutions may be a novel and effective cardioprotective strategy. Nevertheless, the short half-life of angiotensin-(1-7) and angiotensin-(1-9) limits their therapeutic use in a clinical setting. This problem may be overcome by the synthesis of more stable analogs [113]. Alternatively, pharmacological activation of ACE2 -the enzyme that produces these peptides- may also be a promising strategy to enhance the protection induced by cardioplegia. Preclinical studies have shown that treatment with diminazene aceturate -an ACE2 activator- can improve left ventricular function and reduce the incidence of arrhythmias after ischemia/reperfusion injury [132, 133]. However, diminazene aceturate is not used in the clinical arena, given its relevant side effects and toxicity. Therefore, the development of safer drugs targeting the activation of ACE2 is required before this approach can be translated from bench to bedside.

## 5 PERSPECTIVES

The choice of the optimal cardioplegic solution as well as its delivery mechanism remain controversial issues in the field of cardiovascular surgery [34, 134]. Each surgery center chooses the cardioplegic solution to be used in all their patients, both acute and chronic, based on their experience. Moreover, most of the studies have only focused on changing the concentrations and forms of administration of the components [34]. And few institutions have devoted themselves to studying the effect of adjuvant components with the potential to enhance the cardioprotective effect of cardioplegic solutions [33, 135].

The cardioplegic solutions of St. Thomas, Burckberg, Del Nido and Custodiol are the most widely used in cardiovascular surgery because they have generated good results [20, 21]. However, these cardioplegias have continually faced the challenge of offering greater cardioprotection in elderly patients or those with underlying cardiovascular pathologies such as left ventricular hypertrophy, a pathology secondary to processes of hypertension or myocardial infarction and characterized by changes morphological and functional changes in cardiomyocytes that make them more susceptible to death during periods of I/R [136]. Indeed, hypertrophic hearts result in functional impairment when exposed to ischemia and cardioplegic arrest compared to control hearts [137].

## CONCLUSION

Our group has been studying the effect of the Ang-(1-9) and Ang-(1-7) as components of the Del Nido cardioplegia in cardiomyocytes and isolated rat hearts subjected to I/R. Our group is interested in evaluating the effect on cell death and metabolism of the cardiomyocyte, cells responsible for contractile capacity, as well as the role that these new peptides may play in the recovery of the heart after coming out of cardioplegic arrest and myocardial ischemia in isolated rat hearts. The associated use of peptides Ang-(1-9) and Ang-(1-7) could be a potential cardioprotective approach to use during cardioplegia.

In recent years, the exploration of novel adjuvant components for cardioplegic solutions has created increasing attention within the field of cardiovascular surgery. The quest for enhanced cardioprotection, particularly in populations with specific cardiovascular challenges, such as elderly patients or those with pre-existing conditions like left ventricular hypertrophy, underscores the urgency of this pursuit. These conditions present unique physiological intricacies, rendering conventional cardioplegic solutions less effective in safeguarding the myocardium during periods of ischemia and reperfusion. The introduction of peptides Ang-(1-9) and Ang-(1-7) into the Del Nido cardioplegia represents a promising frontier in this endeavor. Preliminary studies within our research group have shown encouraging results for the potential of these peptides to mitigate cell death and restore metabolic function in cardiomyocytes. This innovative approach not only holds promise for improving outcomes in cardiovascular surgery but also opens avenues for further investigation into the interplay between adjuvant components and cardioprotection.

## Figures and Tables

**Fig. (3):**
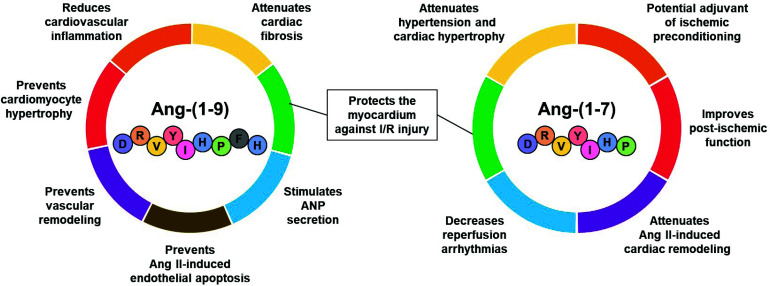
Cardioprotective effects of Ang-(1-9) and Ang-(1-7).

**Table 1:** Composition of the cardioplegic solutions.

**Cardioplegia**	**Composition**
Crystalloid Cardioplegia	Potassium, 19.6 mmol/L; magnesium, 16.7 mmol/L; calcium, 2.0 mmol/L; sodium, 128.0 mmol/L; procaine hydrochloride, 1.0 mmol/L; acetate, 29.4 mmol/L, and chloride 157.8 mmol/L (pH 6.3; temperature, 4°C – 8°C) [130].
Cold Crystalloid Cardioplegia	Glucose 400 mg/dl; sodium 122 mEq/L; potassium 28 mEq/L; chloride 104 mEq/L; bicarbonate 23 mEq/L; total calcium 10.2 mg/dl; ionized calcium 4.87 mg/dl; pH 7.54; Po_2_ 250 mm Hg; osmolarity 366 mOsm/Kg; total protein 2.8 g/dL and albumin 1.7 gr/dL [131].
St. Thomas Hospital Cardioplegia, Solution N°1 (McCarthy)	Sodium Chloride 144.0 mmol/L; potassium chloride 20.0 mmol/L; magnesium chloride 16.0 mmol/L; calcium chloride 2.4 mmol/L; procaine hydrochloride 1.0 mmol/L; pH 5.5 – 7.0; osmolarity 300 – 320 mOsm/kg H_2_O [45].
St. Thomas Hospital Cardioplegia N°2 (Plegisol)	Sodium Chloride 110.0 mmol/L; potassium chloride 16.0 mmol/L; magnesium chloride 16.0 mmol/L; calcium chloride 1.2 mmol/L; Sodium bicarbonate 10.0 mmol/L; pH 7.8; osmolarity 285 – 300 mOsm/kg H_2_O [45].
Blood Cardioplegia	Potassium, 21.5 mmol/L; magnesium, 18.2 mmol/L; calcium, 2.2 mmol/L; sodium, 145.1 mmol/L; procaine hydrochloride, 1.1 mmol/L; acetate, 6.5 mmol/L; chloride, 154.9 mmol/L; and hydrogen carbonate, 28.9 mmol/L (pH 7.4; temperature, 4°C – 8°C) [130].
Del Nido Cardioplegia	The Del Nido solution contains 1 L Plasma-Lyte A base solution to which the following are added: mannitol 20% 16.3 mL; magnesium sulfate 50% 4 mL; Sodium bicarbonate 8.4% 13 mL; potassium chloride (2 mEq/mL) 13 mL and lidocaine 1% 13 mL [113].
Cardi-Braun’s Cardioplegia	For 500 ml the solution contains: Trometamol 4,281 g; Sodium Citrate 3,098 g; Citric acid monohydrate 0,386 g; Sodium dihydrogen phosphate dehydrate 0,295 g; Potassium chloride 1,1709 g; Glucose monohydrate 20,2765 g; Aspartic acid 3,949 g; Glutamic acid 4,400 g, temperature (4-8°C) [132].

## LIST OF ABBREVIATIONS

ACE2: Angiotensin Converting Enzyme 2
ADKC: Activators of ATP-dependent K^+^ Channels
Ang-(1-7): Angiotensin-(1-7)
Ang-(1-9): Angiotensin-(1-9)
AT2R: Angiotensin II Type 2 Receptor
ATP: Adenosine Triphosphate
CABG: Coronary Artery Bypass Grafting
cAMP: Cyclic Adenosine Monophosphate
CAPE: Caffeic Acid Phenethyl Ester
cGMP: Cyclic Guanosine Monophosphate
CPB: Cardiopulmonary Bypass
Drp1: Dynamin related Protein 1
H_2_S: Hydrogen Sulfide.
HIF-1: Hypoxia-Inducible Factor
HTK: Brettschneider’s Histidine-tryptophan-Ketoglutarate
I/R: Ischemia/Reperfusion
MasR: Mas Receptor
NO: Nitric Oxide
PDE5: Phosphodiesterase-5
RAS: Renin-Angiotensin System
ROS: Reactive Oxygen Species
STH: St Thomas' Hospital
STH-1: STH Solution 1
STH-2: STH Solution 2
VEGF: Vascular Endothelial Growth Factor
